# Cross-cultural adaptation and validation of the Chinese version of the Reward Deficiency Syndrome Questionnaire (RDSQ-29)

**DOI:** 10.3389/fpsyg.2026.1929198

**Published:** 2026-10-08

**Authors:** Chenyan Zhao, Xia Chen, Jieting Chen, Xiaojing Zhang, Baifa Zu

**Affiliations:** School of Nursing, Shihezi University, Shihezi, Xinjiang, China

**Keywords:** addictive behaviors, Chinese adaptation, college students, measurement invariance, reward deficiency syndrome, validation

## Abstract

**Objective:**

This study aimed to translate the RDSQ-29 into Chinese and evaluate its reliability, validity, and cross-gender measurement invariance among Chinese university students.

**Methods:**

A convenience sample of 1,628 university students in Xinjiang, China, was recruited for this cross-sectional study. The translation followed the Brislin model. Psychometric evaluations included item analysis, exploratory factor analysis (EFA) conducted in one subsample (*n* = 883), and confirmatory factor analysis (CFA) conducted in another subsample (*n* = 745). Criterion validity was assessed by examining correlations with the Barratt Impulsiveness Scale-Brief (BIS-Brief) and the Internet Gaming Disorder Scale-Short Form (IGDSF-9). Internal consistency and test–retest reliability were evaluated, and multi-group CFA was used to assess measurement invariance across genders.

**Results:**

The Chinese RDSQ-29 retained the original five-factor structure (activity, lack of sexual satisfaction, social concerns, risk-seeking behavior, and general reward deficiency). CFA demonstrated excellent model fit (χ^2^/df = 2.790, comparative fit index [CFI] = 0.968, Tucker–Lewis index [TLI] = 0.963, root mean square error of approximation [RMSEA] = 0.049). The total score showed strong positive correlations with the BIS-Brief (*r* = 0.660, *p* < 0.001) and the IGDSF-9 (*r* = 0.398, *p* < 0.001). Internal consistency was excellent (total scale α = 0.975; subscales α values = 0.819–0.957), and test–retest reliability was high (intraclass correlation coefficient [ICC] = 0.924). Full measurement invariance across genders was established (ΔCFI ≤ 0.003, ΔRMSEA ≤ 0.001).

**Conclusion:**

The Chinese version of the RDSQ-29 demonstrated good reliability and validity and is suitable for assessing reward deficiency syndrome (RDS) among Chinese university students.

## 1 Introduction

Reward deficiency syndrome (RDS) was first proposed by Blum and colleagues in 1995. It is a neurogenetic disorder primarily characterized by dysfunction in the brain’s dopamine-mediated reward pathways (Blum et al., 2000; Blum et al., 2022a). The core feature of this syndrome is a diminished ability to experience satisfaction in daily activities. This may drive individuals to seek high-intensity stimuli, such as substance abuse or behavioral addictions, to compensate for these deficits (Volkow et al., 2017; Blum et al., 2022c). Consequently, this condition may increase an individual’s susceptibility to addiction and related mental disorders (Weber et al., 2025; Calderaro et al., 2025).

RDS is closely associated with various psychiatric and behavioral disorders, including substance use disorder (Lopez-Leon et al., 2021), pathological gambling (Lebedev et al., 2025), attention deficit hyperactivity disorder (ADHD) (Madhusoodanan, 2026), and depression (Halahakoon et al., 2020). Early research primarily relied on biomarkers, such as the genetic addiction risk score (GARS) for RDS assessment (Dennen et al., 2022). Kotyuk et al. (2022) developed the self-reported reward deficiency syndrome questionnaire-29 (RDSQ-29), which provided a practical tool for assessing RDS in the general population. However, subsequent validation studies in different languages (e.g., Hebrew) revealed variations in the scale’s factor structure, suggesting that its dimensions may be influenced by cultural factors (Kotyuk et al., 2025; Efrati et al., 2025).

University students represent a high-risk group for addictive behaviors (e.g., gaming addiction and excessive social media use) (Ren et al., 2025; Li et al., 2024). Given the significant cultural differences between Eastern and Western societies in reward processing mechanisms and social norms, rigorous cross-cultural adaptation and validation of the RDSQ-29 in Chinese samples are particularly necessary. Specifically, concepts such as sexual satisfaction, risk-taking behavior, and social conformity carry unique connotations and may be associated with cultural sensitivities in Chinese culture, requiring careful handling during scale revision to ensure conceptual equivalence and respondent comfort. Additionally, given the notable gender differences in addictive behaviors and reward sensitivity (Van Borkulo et al., 2023; Liu et al., 2024), examining whether the scale exhibits an equivalent measurement structure across different gender groups is crucial for ensuring fairness and validity in group comparisons.

Accordingly, the present study aimed to: (1) translate and culturally adapt the RDSQ-29 into Chinese; (2) evaluate its psychometric properties, including structural validity, criterion validity, internal consistency, and test–retest reliability, in a Chinese university student sample; and (3) assess measurement invariance across genders. Achieving these objectives may contribute to the development of a culturally appropriate and psychometrically sound assessment tool for evaluating RDS in the Chinese context. This instrument could facilitate cross-cultural comparisons and etiological research on reward deficiency, serve as an early screening tool for school nurses and mental health professionals, and provide a theoretical foundation for developing precision nursing interventions targeting the reward system in high-risk student populations.

## 2 Methods

### 2.1 Study design and participants

A cross-sectional, survey-based design was employed. A convenience sample of 1,628 full-time undergraduate students was recruited from a comprehensive university in Xinjiang, China. This university was selected because of its diverse student body, which represented various ethnic and regional backgrounds within China, thereby providing a suitable preliminary sample for scale validation in a non-Western context. Data collection occurred between January 2026 and April 2026. The inclusion criteria were as follows: (1) being aged 18–25 years; (2) being enrolled as a full-time undergraduate student; and (3) providing informed consent. The exclusion criteria included a history of severe mental illness or cognitive impairment.

*Sample size determination:* Following established guidelines for factor analysis (Costello and Osborne, 2005), we adopted a conservative subject-to-variable ratio of 10:1. The study included 29 scale items and 58 analytical variables (29 items, five subscale dimensions, and demographic variables), yielding a minimum required sample size of approximately 580 participants. After allowing for 20% invalid responses, the target sample size was approximately 696 participants. A total of 1,628 valid questionnaires were ultimately collected, exceeding the minimum required sample size and providing adequate statistical power for both exploratory factor analysis (EFA) and confirmatory factor analysis (CFA).

*Subsample allocation:* The total sample was randomly divided into two independent, non-overlapping subsamples using the random selection function in SPSS. Sample 1 (*n* = 883) was used for item analysis and exploratory factor analysis (EFA), with a subject-to-item ratio of approximately 30:1. Sample 2 (*n* = 745) was used for confirmatory factor analysis (CFA), criterion validity, internal consistency, and measurement invariance, with a subject-to-item ratio of approximately 26:1. This random division allowed the factor structure identified in Sample 1 to be cross-validated in Sample 2 (Fabrigar et al., 1999).

*Test*–*retest subsample:* Four weeks after the initial survey, 250 participants were randomly selected from Sample 2 for a retest, and 214 valid questionnaires were returned. This subsample was nested within Sample 2; therefore, the same participants contributed to both cross-sectional validity and longitudinal reliability analyses (Mokkink et al., 2018; Kalfoss, 2019).

### 2.2 Translation and cultural adaptation

The translation and cultural adaptation process followed Brislin’s model (Brislin, 2007; Beaton et al., 2000). A detailed comparison of the translations (Table A1) is provided in the Supplementary Files. Two bilingual experts (one psychologist and one linguist) independently translated the original English scale into Chinese. The two translations were synthesized into a preliminary version by the research team. This version was then back-translated into English by two other bilingual experts who were blinded to the original scale. A panel comprising three clinical psychologists, two psychometricians, and one bilingual linguist reviewed all versions. Discrepancies were discussed until consensus was reached on semantic, idiomatic, experiential, and conceptual equivalence.

Subsequently, an expert consultation was conducted with five additional experts in clinical psychology and addictive behaviors to evaluate the content validity and cultural appropriateness of each item. Based on the experts’ feedback, minor adjustments were made. Finally, a cognitive pre-test (pilot test) involving 30 students from the target population was conducted to assess item comprehension, clarity (Kalfoss, 2019; Flake and Fried, 2020), relevance, and approximate completion time, resulting in final wording refinements.

## 3 Measures

### 3.1 Questionnaire

#### 3.1.1 Demographic questionnaire

This questionnaire was a self-developed information form designed to collect participants’ sociodemographic characteristics, including gender, age, educational level (grade), and family background (e.g., parental educational attainment and family income).

Chinese Version of the Reward Deficiency Syndrome Questionnaire-29 (RDSQ-29).

The original 29-item Reward Deficiency Syndrome Questionnaire (RDSQ-29) was developed by Blum et al. to assess symptoms of reward deficiency (Kotyuk et al., 2022). The Chinese version of the RDSQ-29 was translated and validated for use in Chinese populations. The scale comprises 29 items distributed across five dimensions: activity, lack of sexual satisfaction, social concerns, risk-seeking behavior, and general reward deficiency. Participants responded using a 4-point Likert scale (1 = Strongly Disagree to 4 = Strongly Agree), with higher total scores indicating greater reward deficiency severity. In the present study, the Cronbach’s α coefficient for the Chinese version of the RDSQ-29 was 0.975.

#### 3.1.2 Barratt Impulsiveness Scale-Brief (BIS-Brief), Chinese version

An 8-item Barratt Impulsiveness Scale-Brief (BIS-Brief) measures impulsivity. The original scale was developed by Steinberg et al. to provide a rapid assessment of impulsivity traits (Steinberg et al., 2013). The Chinese version used in this study has been validated in prior research (Luo et al., 2020). This unidimensional scale measures general impulsivity. All items are rated on a 4-point Likert scale (1 = rarely/never to 4 = almost always/always), with higher scores reflecting higher levels of impulsivity. In the present study, the Cronbach’s α coefficient for the BIS-Brief was 0.736.

#### 3.1.3 Internet Gaming Disorder Scale–Short Form (IGDSF-9) Chinese version

The original 9-item Internet Gaming Disorder Scale–Short Form was developed by Pontes and Griffiths based on the DSM-5 criteria to assess symptoms of Internet gaming disorder (Pontes and Griffiths, 2015). The Chinese version used in this study has demonstrated good psychometric properties (Yam et al., 2019). This unidimensional scale assesses the severity of Internet gaming disorder symptoms over the past year. Items are rated on a 5-point Likert scale (1 = never to 5 = almost always). The total score is computed by summing the scores for all nine items, with a possible range of 9–45; higher scores indicate greater symptom severity. In the present study, Cronbach’s α for the Internet Gaming Disorder Scale-Short Form (IGDSF-9) was 0.824.

### 3.2 Data collection

Data were collected online via the “Wenjuanxing” platform, with supplementary paper-based surveys administered in classrooms to increase the participation rate (Wu et al., 2022; Fan and Yan, 2010). Participants provided informed consent electronically or in writing before completing the survey. Incomplete responses and responses with unrealistically short completion times (defined as less than one-third of the median completion time) were excluded from analysis (Leiner, 2019).

### 3.3 Statistical analysis

Data were analyzed using SPSS 27.0, AMOS 29.0, and Mplus 8.3.

*Item analysis:* The critical ratio (CR) and corrected item-total correlations were calculated. Items with a CR value greater than 3.0 and an item-total correlation greater than 0.30 were considered satisfactory (Alhadabi and Karpinski, 2020).

*Structural validity:* Given the theoretical underpinnings of the RDS construct, exploratory factor analysis (EFA) was performed on Sample 1 using principal axis factoring with varimax rotation (Fabrigar et al., 1999). The number of factors was determined based on eigenvalues greater than 1, inspection of the scree plot, and theoretical interpretability. Confirmatory factor analysis (CFA) was conducted on Sample 2 using maximum likelihood estimation. In the present study, we specified a correlated five-factor model comprising activity, lack of sexual satisfaction, social concerns, risk-seeking behavior, and general reward deficiency. This specification differs from the bifactor model used in the original development study (Kotyuk et al., 2022), in which all items loaded onto a general factor and simultaneously onto a specific factor. Model fit was evaluated using multiple indices and contemporary benchmarks: χ^2^/df < 3, comparative fit index (CFI) > 0.95, Tucker–Lewis index (TLI) > 0.95, root mean square error of approximation (RMSEA) < 0.06, and standardized root mean square residual (SRMR) < 0.08 (Kelava, 2016; Raykov et al., 2025).

*Criterion validity:* Pearson correlation coefficients were calculated between the total and subscale scores of the RDSQ-29 and total scores of the BIS-Brief and IGDSF-9 (Cohen, 1988). Correlation magnitudes were interpreted as small (*r* ~ 0.10), medium (*r* ~ 0.30), and large (*r* ~ 0.50).

*Reliability:* Internal consistency was assessed using Cronbach’s α, with values of ≥ 0.70 considered acceptable for subscales and values of ≥ 0.80 considered acceptable for the total scale. Test–retest reliability over 4 weeks was evaluated using the intraclass correlation coefficient (ICC; a two-way mixed-effects model for absolute agreement), with values > 0.75 indicating good reliability (Mokkink et al., 2018).

*Measurement invariance across genders:* Multi-group CFA was employed to test four sequential levels of invariance using Sample 2: configural invariance (the same factor structure), metric invariance (equal factor loadings), scalar invariance (equal item intercepts), and strict invariance (equal residual variances). Invariance was assessed using changes in robust fit indices (ΔCFI, ΔTLI, ΔRMSEA, and ΔSRMR) between nested models. Following current guidelines (Marsh et al., 2019), invariance was supported if ΔCFI ≤ 0.010 and ΔRMSEA ≤ 0.015, supplemented by ΔSRMR ≤ 0.030 for metric invariance and ΔSRMR ≤ 0.015 for scalar and strict invariance.

## 4 Results

### 4.1 Sample characteristics

Sample 1 (used for item analysis and exploratory factor analysis) was selected from an undergraduate population at a university in Xinjiang using a convenience sampling method. A total of 900 questionnaires were distributed, and 883 valid questionnaires were collected (effective response rate: 98%). The sample comprised 315 men (35.7%) and 568 women (64.3%); ages ranged from 18 to 25 years, with a mean age of 20.57 ± 1.681 years. Of the participants 188 were first-year students (21.3%), 261 were second-year students (29.6%), 287 were third-year students (32.5%), 116 were fourth-year students (13.1%), and 31 were fifth-year students (3.5%). A total of 742 participants were from urban areas (84.0%), whereas 141 were from rural areas (16.0%). Additionally, 510 participants were only children (57.8%), whereas 373 were not only children (42.2%).

Sample 2 (used for confirmatory factor analysis, criterion-related validity assessment, internal consistency analysis, and measurement invariance across genders) was also selected from an undergraduate population at a university in Xinjiang using a convenience sampling method. A total of 850 questionnaires were distributed, and 745 valid questionnaires were collected (effective response rate: 87%). The sample comprised 347 men (46.6%) and 398 women (53.4%). Participants’ ages ranged from 18 to 25 years, with a mean age of 20.64 ± 1.711 years. Of the participants, 140 were first-year students (18.8%), 228 were second-year students (30.6%), 244 were third-year students (32.8%), 115 were fourth-year students (15.4%), and 18 were fifth-year students (2.4%). A total of 622 participants were from urban areas (83.5%), whereas 123 from rural areas (16.5%). Additionally, 434 participants were only children (58.3%), whereas 311 were not only children (41.7%).

Sample 3 (used for test–retest reliability analysis) was formed by randomly selecting 250 participants from Sample 2 4 weeks after the initial survey. A total of 214 valid questionnaires were ultimately obtained (effective response rate: 85%). Of the participants, 46 were first-year students (21.5%), 67 were second-year students (31.3%), 63 were third-year students (29.4%), 34 were fourth-year students (15.9%), and four were fifth-year students (1.9%). Participants’ ages ranged from 18 to 25 years, with a mean age of 20.66 ± 1.838 years.

### 4.2 Item analysis

All 29 items demonstrated satisfactory discriminatory power. Item-total correlations ranged from 0.245 to 0.765 (*p* < 0.001), and independent-samples t-tests comparing the high-score and low-score groups (the upper and lower 27% of participants, respectively) were all significant (*p* < 0.001) (Table 1). Removal of any item did not increase the Cronbach’s α coefficient of the total scale.

**Table 1:** Item discrimination analysis.

Items	*t*	*r*
1	13.59***	0.43***
2	11.04***	0.40***
3	12.52***	0.42***
4	10.67***	0.40***
5	9.16***	0.34***
6	10.05***	0.37***
7	10.41***	0.37***
8	10.58***	0.38***
9	6.84***	0.27***
10	5.25***	0.24***
11	16.32***	0.51***
12	17.78***	0.53***
13	15.21***	0.49***
14	17.80***	0.52***
15	13.61***	0.46***
16	21.02***	0.68***
17	25.60***	0.72***
18	21.45***	0.67***
19	23.48***	0.67***
20	27.13***	0.72***
21	28.34***	0.73***
22	25.03***	0.73***
23	23.36***	0.70***
24	24.79***	0.70***
25	27.33***	0.72***
26	27.41***	0.70***
27	30.19***	0.74***
28	30.21***	0.76***
29	27.28***	0.74***

***p < 0.001.

### 4.3 Exploratory factor analysis

For Sample 1, the Kaiser–Meyer–Olkin (KMO) measure was 0.924, and Bartlett’s test of sphericity was significant (χ^2^ = 18,914.214, *p* < 0.001). Principal component analysis identified five factors with eigenvalues >1, which collectively explained 70.26% of the variance. Factor loadings ranged from 0.717 to 0.932, consistent with the original five-factor structure (Table 2). This five-factor solution explained 70.258% of the total variance, and inspection of the scree plot further supported the retention of five factors (Figure 1).

**Table 2:** Complete exploratory factor analysis results (*n* = 883).

Items	Factor 1	Factor 2	Factor 3	Factor 4	Factor 5
1	0.812				
2	0.897				
3	0.883				
4	0.851				
5	0.779				
6		0.907			
7		0.932			
8		0.902			
9			0.895		
10			0.895		
11				0.811	
12				0.801	
13				0.781	
14				0.811	
15				0.769	
16					0.729
17					0.750
18					0.743
19					0.717
20					0.792
21					0.791
22					0.826
23					0.786
24					0.786
25					0.823
26					0.775
27					0.813
28					0.816
29					0.799

Factor Statistics: Factor 1 (Activity): Eigenvalue = 3.667;variance explained = 12.644%. Factor 2 (Lack of Sexual Satisfaction): Eigenvalue = 2.259; variance explained = 7.791%. Factor 3 (Social Concerns): Eigenvalue = 1.463; variance explained = 5.045%. Factor 4 (Risk-Seeking Behavior): Eigenvalue = 2.757; variance explained = 9.508%. Factor 5 (General Reward Deficiency): Eigenvalue = 10.229; variance explained = 35.271%.

**Figure 1:**
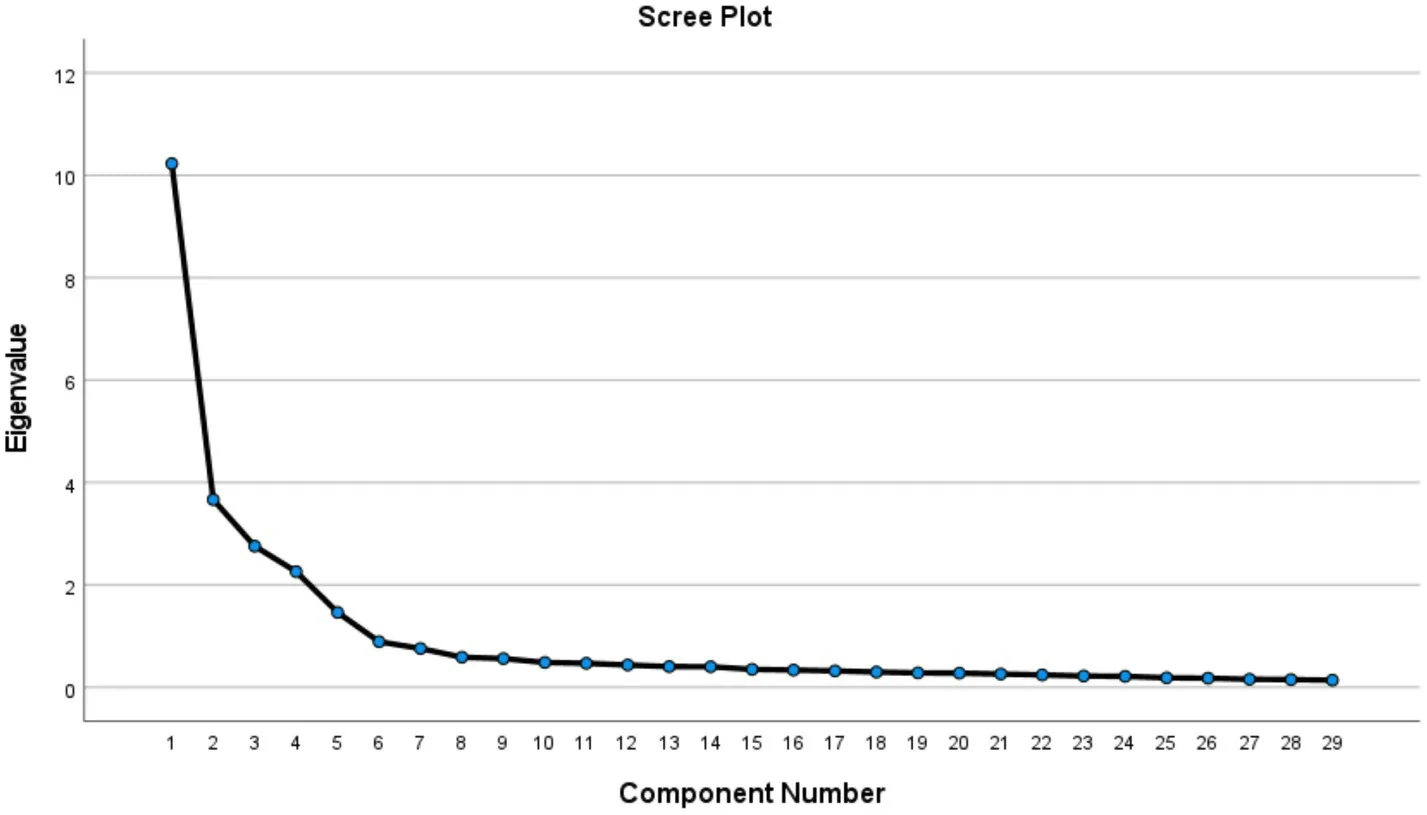
This scree plot displays component numbers (*x*-axis) against eigenvalues (*y*-axis), which represent explained variance. The curve drops sharply after the first component and levels off at the fifth, where the eigenvalue approaches 1. The distinct at component 5, followed by a flat tail, confirms that extracting five sub-factors is appropriate for explaining the majority of the variance.

### 4.4 Confirmatory factor analysis

CFA conducted on Sample 2 confirmed the five-factor structure and demonstrated excellent model fit: χ^2^ = 982.063, χ^2^/df = 2.790, CFI = 0.968, TLI = 0.963, RMSEA = 0.049 (90% CI: 0.046–0.053), and SRMR = 0.029. All standardized factor loadings were significant and exceeded 0.70 (range: 0.726–0.944). The complete CFA results are shown in Figure 2.

**Figure 2:**
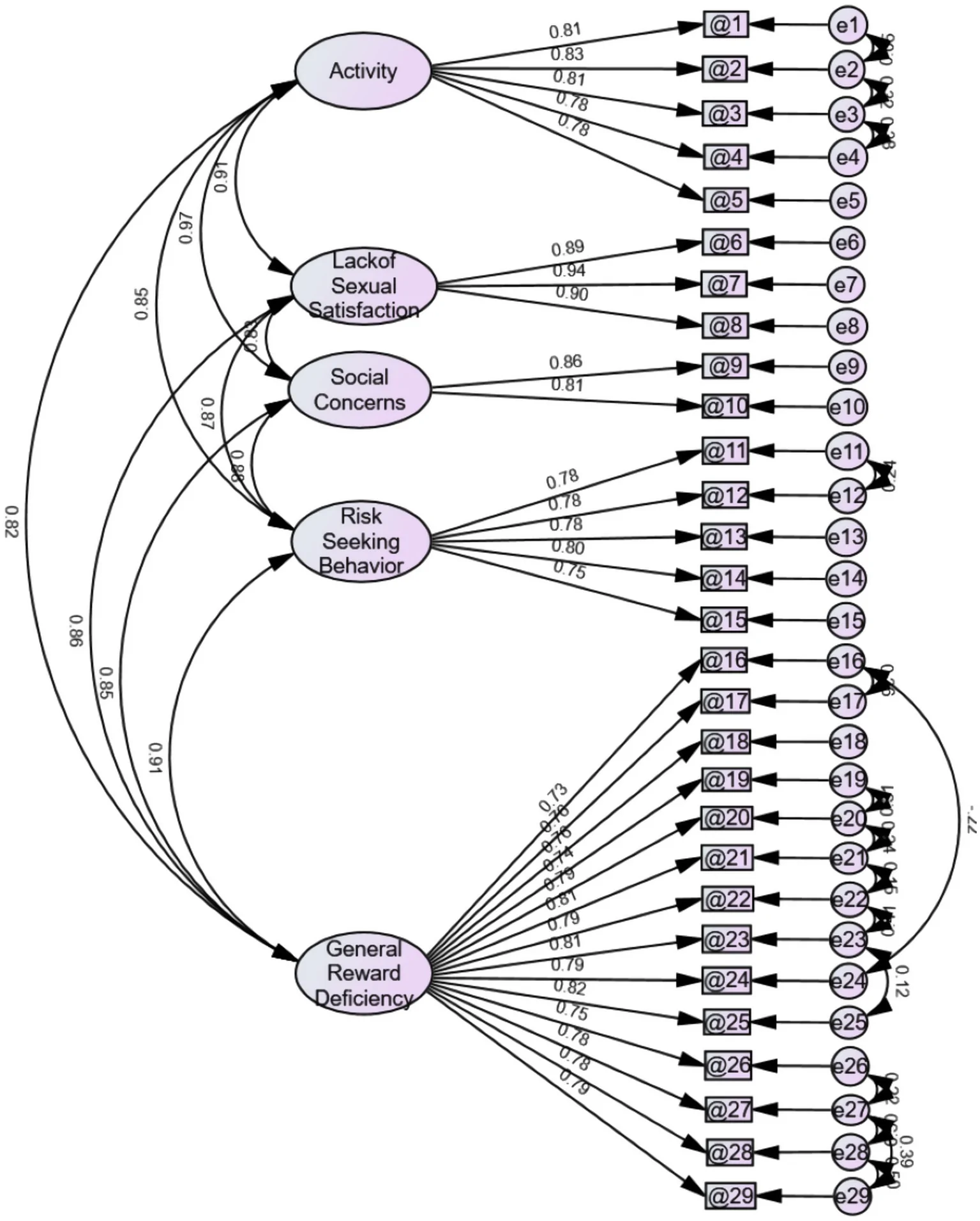
This structural equation model examines the psychological links among Activity, Lack of Sexual Satisfaction, Social Concerns, Risk Seeking Behavior, and General Reward Deficiency. It comprises 5 latent variables and 29 observed indicators. Path coefficients reveal that Activity strongly predicts Lack of Sexual Satisfaction (0.97) and Reward Deficiency (0.91), while significant chained mediation effects exist among all variables.

### 4.5 Criterion validity

The total RDSQ-29 score showed a strong positive correlation with the BIS-Brief (*r* = 0.660, *p* < 0.001) and a moderate positive correlation with the IGDSF-9 (*r* = 0.398, *p* < 0.001). All subscales also showed significant positive correlations with both criterion measures (all *p* < 0.001) (Table 3).

**Table 3:** Correlations between Chinese RDSQ-29 and criterion measures.

Variables	**BIS-brief (Total)**	**IGDSF-9**
Activity	0.616***	0.435***
Lack of sexual satisfaction	0.618***	0.404***
Social concerns	0.535***	0.334***
Risk seeking behavior	0.651***	0.356***
General reward deficiency	0.596***	0.347***
RDSQ-29 total score	0.660***	0.398***

***p < 0.001.

### 4.6 Reliability analysis

Internal consistency was excellent: Cronbach’s α was 0.975 for the total scale, ranging from 0.819 to 0.957 across the subscales. Test–retest reliability over 4 weeks was high, with an ICC of 0.924 for the total score and subscale ICCs ranging from 0.781 to 0.959 (Table 4).

**Table 4:** Internal consistency and test–retest reliability of the Chinese RDSQ-29.

Variables	Cronbach’s α	ICC
Activity	0.908	0.916
Lack of sexual satisfaction	0.934	0.942
Social concerns	0.819	0.781
Risk seeking behavior	0.888	0.889
General reward deficiency	0.957	0.959
RDSQ-29 total	0.975	0.924

### 4.7 Measurement invariance across genders

Multi-group CFA was conducted to assess measurement invariance across genders. As shown in Table 5, the configural model demonstrated acceptable fit, indicating that the five-factor structure was supported in both male and female groups. When factor loadings (metric invariance), intercepts (scalar invariance), and residuals (strict invariance) were constrained to be equal across groups, the changes in model fit indices (ΔCFI and ΔRMSEA) were negligible and well below the recommended thresholds for invariance (i.e., ΔCFI ≤ 0.010 and ΔRMSEA ≤ 0.015). These findings support full measurement invariance of the Chinese RDSQ-29 across genders, suggesting that the scale has comparable measurement properties in male and female university students. Therefore, comparisons of observed scores between these groups may be conducted under the assumption of measurement invariance.

**Table 5:** Tests of measurement invariance across genders.

Model	χ^2^	df	CFI	RMSEA	△CFI	△RMSEA
Configural	2730.071	734	0.902	0.085		
Metric	2749.993	758	0.902	0.084	0.000	−0.001
Scalar	2767.626	782	0.903	0.083	0.001	−0.001
Strict	2844.189	811	0.900	0.082	−0.003	−0.001

## 5 Discussion

This study completed the translation and psychometric validation of the Chinese version of the RDSQ-29. The validation results supported its original five-factor structure, and the scale demonstrated strong internal consistency, high test–retest reliability, and good criterion validity. Notably, the scale exhibited full measurement invariance across genders, supporting its use in between-group comparisons.

### 5.1 Comparison with existing literature

The five-factor correlated structure identified in the present study is broadly consistent with the theoretical construct of Reward Deficiency Syndrome (RDS) and with the factor content reported in the original development study (Kotyuk et al., 2022). However, a key methodological difference should be noted. The original RDSQ-29 was developed within a bifactor framework, in which all items loaded onto a general reward deficiency factor and simultaneously on one of four specific factors: lack of sexual satisfaction, activity, social concerns, and risk-seeking behavior. In that study, the general factor explained 68–69% of the common variance, and the omega-hierarchical coefficient for the general factor ranged from 0.90 to 0.94, supporting the interpretation of a predominantly unidimensional general reward deficiency construct. Subsequent cross-cultural validations, including the Turkish version (Peker et al., 2025) and the Hebrew version (Kotyuk et al., 2025), also adopted a bifactor model or initially evaluated a bifactor CFA model.

In the present study, we tested a five-factor correlated model rather than a bifactor model. In this model, General Reward Deficiency represents one of five correlated factors rather than a global factor onto which all items load simultaneously. Conceptually, this factor corresponds to the general factor in the original bifactor model because it captures the broad tendency toward diminished reward responsiveness, while the other four factors correspond to the specific factors in the original model. The model demonstrated good model fit (CFI = 0.968, TLI = 0.963, RMSEA = 0.049, and SRMR = 0.029), supporting the adequacy of the five-factor content structure in Chinese university students. However, because our CFA did not include cross-loadings of all items onto a general factor, the present study does not provide a direct statistical test of the bifactor structure. Future studies should directly compare the bifactor model and the five-factor correlated model in Chinese samples, and report ECV, omega-hierarchical, and PUC indices, as in the original development study and the Turkish validation (Kotyuk et al., 2022; Peker et al., 2025).

It is worth noting that the factor content may be particularly relevant to the Chinese cultural context. For instance, items in the Social Concerns factor, which reflect concerns expressed by family members and friends regarding an individual’s lifestyle, may highlight the cultural emphasis on familial harmony and social evaluation in collectivist societies. This observation may support the relevance of the cultural adaptation (Xu et al., 2019; Pang, 2022). The strong correlation between scale scores and impulsivity, as measured by the BIS-Brief, is consistent with the proposed relationship between RDS and inhibitory control. Meanwhile, the moderate correlation with Internet Gaming Disorder, as measured by the IGDSF-9, suggests shared features related to reward-processing deficits (Hildebrandt et al., 2023; Sobih et al., 2025). These findings indicate that the RDSQ-29 may be useful for investigating factors associated with behavioral addictions (Blum et al., 2022b).

Of particular interest are the confirmatory factor analysis (CFA) fit indices and the results of the measurement invariance analysis. The initial CFA demonstrated good model fit (CFI = 0.968), although the absolute fit indices in the multi-group CFA conducted for invariance testing were below commonly used thresholds for excellent fit (e.g., CFI = 0.902). This pattern may occur in large and complex multi-group models, in which larger samples sizes can increase sensitivity to minor model misspecifications (Shi and Maydeu-Olivares, 2019). Additionally, the high standardized factor loadings (0.726–0.944) suggest strong associations between the items and their respective factors, although they may also indicate potential item redundancy or common method bias. The primary evidence for measurement invariance is based on changes in fit indices between nested models (ΔCFI, ΔRMSEA), all of which were within the established thresholds (ΔCFI ≤ 0.003, ΔRMSEA ≤ 0.001). Thus, we conclude that full measurement invariance is supported. However, the observed fit patterns also suggest the potential for developing a more concise short-form version in the future research.

The establishment of full measurement invariance is a significant contribution. It ensures that any observed gender differences in future studies using this scale reflect true group differences in RDS traits rather than measurement bias, thereby enhancing the validity of cross-group comparisons (Putnick and Bornstein, 2016; Svetina et al., 2019).

### 5.2 Practical and research implications

The Chinese version of the RDSQ-29 provides researchers and potentially clinicians with a reliable and valid tool for assessing reward deficiency among Chinese university students. The scale can be used for early identification of individuals at high risk of addictive behaviors, etiological research, and evaluating the effectiveness of targeted interventions aimed at enhancing natural reward sensitivity.

### 5.3 Limitations

This study has several limitations. First, the use of a convenience sample from a single university and region may limit the generalizability of findings to the broader Chinese student population. Future studies should include more geographically diverse and nationally representative samples to further evaluate the scale’s generalizability (Roberts et al., 2020; Lilienfeld and Strother, 2020). Second, the cross-sectional design precludes causal inferences and restricts the evaluation of the RDSQ-29’s predictive validity for subsequent addictive behaviors (Rohrer, 2018). Third, while criterion validity has been established, future research could include more objective measures (e.g., behavioral tasks and neuroimaging data) to verify convergent validity. Fourth, all data were derived from self-report measures, which may introduce common method bias and inflate observed correlations between constructs. Fifth, the present study tested a five-factor correlated model rather than the bifactor model used in the original development study (Kotyuk et al., 2022) and in the Turkish validation (Peker et al., 2025). Because our CFA did not include cross-loadings of all items on a general factor, we could not evaluate the proportion of common variance attributable to a general reward deficiency factor (ECV) or the omega-hierarchical coefficient. Future research should directly compare these two model specifications in Chinese samples.

### 5.4 Future research directions

Future studies should employ more diverse and nationally representative samples (Dotson and Duarte, 2020) and adopt longitudinal designs to examine the RDSQ-29’s predictive validity for the onset and progression of addictive behaviors. Additionally, research should explore the scale’s clinical utility and its association with biomarkers of reward system function (Lorenzo-Luaces et al., 2020; Lewandrowski et al., 2025). Building on the current findings, subsequent studies could also focus on developing and validating a short-form version of the scale to enhance practicality without compromising its psychometric properties. Finally, future studies should directly compare the bifactor model and the five-factor correlated model to clarify the dimensionality of the RDSQ-29 in Chinese populations, and should report ECV, omega-hierarchical, and PUC indices to facilitate comparison with the original development study (Kotyuk et al., 2022) and the Turkish validation (Peker et al., 2025).

## 6 Conclusion

This study introduces the Chinese version of the Reward Deficiency Syndrome Questionnaire-29 (RDSQ-29) for the first time and provides systematic evidence for its psychometric properties. The scale demonstrates good reliability and validity among Chinese university students and, crucially, exhibits full measurement invariance across genders, ensuring its suitability for examining gender differences in reward deficiency syndrome traits within the Chinese population. The Chinese version of the RDSQ-29 is an effective tool for assessing reward deficiency syndrome in university students and demonstrates strong applicability following cultural adaptation. This scale holds significant value for clinical screening and etiological research on reward mechanisms related to addictive behaviors, particularly for in-depth studies of this at-risk population.

## Data Availability

The original contributions presented in the study are included in the article/Supplementary material, further inquiries can be directed to the corresponding author.

## References

[ref1] Alhadabi A. Karpinski A. C. (2020). Grit, self-efficacy, achievement orientation goals, and academic performance in university students. Int. J. Adolesc. Youth 25, 519–535. doi: 10.1080/02673843.2019.1679202

[ref2] Beaton D. E. Bombardier C. Guillemin F. Ferraz M. B. (2000). Guidelines for the process of cross-cultural adaptation of self-report measures. Spine 25, 3186–3191. doi: 10.1097/00007632-200012150-00014, 11124735

[ref3] Blum K. Braverman E. R. Holder J. M. Lubar J. F. Monastra V. J. Miller D. . (2000). The reward deficiency syndrome: a biogenetic model for the diagnosis and treatment of impulsive, addictive and compulsive behaviors. J. Psychoactive Drugs 32, 1–112. doi: 10.1080/02791072.2000.10736099, 11280926

[ref4] Blum K. Braverman E. R. Holder J. M. Lubar J. F. Monastra V. J. Miller D. . (2022a). Offering a putative neurobiological “dopamine homeostatic” solution to overcome the perils of the reward deficiency syndrome pandemic: emergence of “precision behavioral management.”. Ann. Transl. Med. 10:1291. doi: 10.21037/atm-2022-67 PMC9816827 36618806

[ref5] Blum K. Elman I. Dennen C. A. McLaughlin T. Thanos P. K. Baron D. . (2022b). “Preaddiction” construct and reward deficiency syndrome: genetic link via dopaminergic dysregulation. Ann Transl Med. 10:1181. doi: 10.21037/atm-2022-32, 36467361 PMC9708493

[ref6] Blum K. McLaughlin T. Bowirrat A. Modestino E. J. Baron D. Gomez L. L. . (2022c). Reward deficiency syndrome (RDS) surprisingly is evolutionary and found everywhere: “is it blowin in the wind”? J Pers Med. 12:321. doi: 10.3390/jpm12020321, 35207809 PMC8875142

[ref7] Brislin R. W. (2007). Comparative research methodology: cross-cultural studies. Int. J. Psychol. 11, 215–229. doi: 10.1080/00207597608247359

[ref8] Calderaro M. L. Cunningham R. M. Quarmley M. Clarkson T. Schmidt H. Cassidy C. M. . (2025). Dopamine function in adolescent social anxiety: insights from neuromelanin-sensitive MRI. J Mood Anxiety Disord. 11:100124. doi: 10.1016/j.xjmad.2025.100124, 40657353 PMC12244091

[ref9] Cohen J. (1988). Statistical power Analysis for the Behavioral Sciences. 2nd Edn Taylor and Francis: Routledge. doi: 10.4324/9780203771587

[ref10] Costello A. B. Osborne J. W. (2005). Best practices in exploratory factor analysis: four recommendations for getting the most from your analysis. Pract. Assess. Res. Eval. 10:7.

[ref11] Dennen C. A. Blum K. Bowirrat A. Thanos P. K. Elman I. Ceccanti M. . (2022). Genetic addiction risk severity assessment identifies polymorphic reward genes as antecedents to reward deficiency syndrome (RDS) hypodopaminergia’s effect on addictive and non-addictive behaviors in a nuclear family. J Pers Med. 12:1864. doi: 10.3390/jpm12111864, 36579592 PMC9694640

[ref12] Dotson V. M. Duarte A. (2020). The importance of diversity in cognitive neuroscience. Ann. N. Y. Acad. Sci. 1464, 181–191. doi: 10.1111/nyas.14268, 31663150

[ref13] Efrati Y. Demetrovics Z. Horváth Z. Griffiths M. D. Potenza M. N. Blum K. . (2025). Seeking satisfaction among Israeli young adults: the roles of gratification, compensation, reward deficiency syndrome, and compulsivity in relation to addictive behaviors. Subst. Abus. Rehabil. 16, 177–191. doi: 10.2147/sar.s527209, 40621506 PMC12229155

[ref14] Fabrigar L. R. Wegener D. T. MacCallum R. C. Strahan E. J. (1999). Evaluating the use of exploratory factor analysis in psychological research. Psychol. Methods 4, 272–299. doi: 10.1037/1082-989x.4.3.272

[ref15] Fan W. Yan Z. (2010). Factors affecting response rates of the web survey: a systematic review. Comput. Hum. Behav. 26, 132–139. doi: 10.1016/j.chb.2009.10.015

[ref16] Flake J. K. Fried E. I. (2020). Measurement schmeasurement: questionable measurement practices and how to avoid them. Adv. Methods Pract. Psychol. Sci. 3, 456–465. doi: 10.1177/2515245920952393

[ref18] Halahakoon D. C. Kieslich K., O’ Driscoll C. Nair A. Lewis G. (2020). Reward-processing behavior in depressed participants relative to healthy volunteers. JAMA Psychiatry 77:1286. doi: 10.1001/jamapsychiatry.2020.2139 PMC7391183 32725180

[ref19] Hildebrandt M. K. Dieterich R. Veredjo-Román J. Endrass T. (2023). Inhibitory control in substance use disorders: disentangling substance use and substance-related problems. Int. J. Psychophysiol. 188:13. doi: 10.1016/j.ijpsycho.2023.05.030

[ref20] Kalfoss M. (2019). Translation and adaption of questionnaires: a nursing challenge. SAGE Open Nurs. 5:237796081881681. doi: 10.1177/2377960818816810, 33415214 PMC7774397

[ref21] Kelava A. (2016). A review of confirmatory factor analysis for applied research (second edition). J. Educ. Behav. Stat. 41, 443–447. doi: 10.3102/1076998616631747

[ref22] Kotyuk E. Demetrovics Z. Urbán R. Czakó A. Blum K. (2025). Psychometric properties of the reward deficiency syndrome questionnaire among a non-clinical sample and its relationship with the characteristics of potentially addictive behaviors. Addict. Behav. Rep. 21:100598. doi: 10.1016/j.abrep.2025.100598 PMC12005299 40248688

[ref23] Kotyuk E. Urbán R. Hende B. Richman M. Magi A. (2022). Development and validation of the reward deficiency syndrome questionnaire (RDSQ-29). J. Psychopharmacol. 36, 409–422. doi: 10.1177/02698811211069102, 35102768

[ref24] Lebedev A. A. Potapkin A. M. Pyurveev S. S. Sizov V. V. Gmiro V. E. Bychkov E. R. . (2025). Blockade of GluA1 AMPA receptors reduces impulsive behavior in a gambling addiction model by modulating extracellular dopamine levels. Rev. Clinic. Pharmacol. Drug Therapy 23, 177–189. doi: 10.17816/rcf646052

[ref25] Leiner D. J. (2019). Too fast, too straight, too weird: non-reactive indicators for meaningless data in internet surveys. Surv Res Methods. 13, 229–248. doi: 10.18148/srm/2019.v13i3.7403

[ref26] Lewandrowski K. U. Bowirrat A. Pinhasov A. Sharafshah A. Gold M. S. Fuehrlein B. . (2025). Dopaminergic homeostatic therapy (DHT™) as a putative anti-addiction seeking intervention and early identification of genetic preaddiction with genetic addiction risk severity (GARS®) screening. Acta Scientiarum Neurol. 8, 70–88. PMC12396177 40896766

[ref27] Li X. Chen X. Zhu Y. Shi X. (2024). Longitudinal associations of social anxiety trajectories with internet-related addictive behaviors among college students: a five-wave survey study. Int. J. Ment. Health Addict. 23, 3467–3483. doi: 10.1007/s11469-024-01300-5

[ref28] Lilienfeld S. O. Strother A. N. (2020). Psychological measurement and the replication crisis: four sacred cows. Can. Psychol. 61, 281–288. doi: 10.1037/cap0000236

[ref29] Liu S. Ren H. Li Y. Liu Y. Fu S. (2024). Gender difference in the onset of adolescent depressive symptoms: a cross-lagged panel network analysis. Res. Child Adolesc. Psychopathol. 53:113–123. doi: 10.1007/s10802-024-01235-4 39215790

[ref30] Lopez-Leon S. Gonzalez-Giraldo Y. Wegman-Ostrosky T. Forero D. A. (2021). Molecular genetics of substance use disorders: an umbrella review. Neurosci. Biobehav. Rev. 124, 358–369. doi: 10.1016/j.neubiorev.2021.01.019, 33556390

[ref31] Lorenzo-Luaces L. Peipert A. De Jesús Romero R. Rutter L. A. Rodriguez-Quintana N. (2020). Personalized medicine and cognitive behavioral therapies for depression: small effects, big problems, and bigger data. Int. J. Cogn. Ther. 14, 59–85. doi: 10.1007/s41811-020-00094-3

[ref32] Luo T. Chen M. Y. Ouyang F. Y. Xiao S. Y. (2020). Reliability and validity of the Chinese version of the brief Barratt impulsiveness scale. Chin. J. Clin. Psychol. 28, 1199–1201.

[ref33] Madhusoodanan J. (2026). Untangling the connection between dopamine and ADHD. Nature. doi: 10.1038/d41586-026-00094-x, 41565954

[ref34] Marsh H. W. Guo J. Dicke T. Parker P. D. Craven R. G. (2019). Confirmatory factor analysis (CFA), exploratory structural equation modeling (ESEM), and set-ESEM: optimal balance between goodness of fit and parsimony. Multivar. Behav. Res. 55, 102–119. doi: 10.1080/00273171.2019.1602503 31204844

[ref35] Mokkink L. B. de Vet H. C. W. Prinsen C. A. C. Patrick D. L. Alonso J. Bouter L. M. . (2018). COSMIN risk of bias checklist for systematic reviews of patient-reported outcome measures. Qual. Life Res. 27, 1171–1179. doi: 10.1007/s11136-017-1765-4, 29260445 PMC5891552

[ref36] Pang X. (2022). FNT: a research about the relationships among conflict styles, face concerns and four individual-level elements: gender, age, occupation and degree of education. E. Mallen. In Proceedings of the 3rd International Conference on Educational Innovation and Philosophical Inquiries (ICEIPI 2022) (pp. 256–263).

[ref37] Peker O. Ugurlu M. Esim A. G. Peker I. Karakas Ugurlu G. Caykoylu A. . (2025). Validation and psychometric evaluation of the Turkish version of the reward deficiency syndrome questionnaire (RDSQ-29). Dusunen Adam 38, 255–265. doi: 10.14744/DAJPNS.2025.00301, 42459588 PMC13289287

[ref38] Pontes H. M. Griffiths M. D. (2015). Measuring DSM-5 internet gaming disorder: development and validation of a short psychometric scale. Comput. Hum. Behav. 45, 137–143. doi: 10.1016/j.chb.2014.12.006

[ref39] Putnick D. L. Bornstein M. H. (2016). Measurement invariance conventions and reporting: the state of the art and future directions for psychological research. Dev. Rev. 41, 71–90. doi: 10.1016/j.dr.2016.06.004, 27942093 PMC5145197

[ref40] Raykov T. Penev S. Zhang B. (2025). Fitting covariance structure models with arbitrary observed distributions: strong convergence of the asymptotically distribution-free estimator. Struct. Equ. Model. 33, 32–38. doi: 10.1080/10705511.2025.2556028

[ref41] Ren Z. Lu H. Zheng P. Lu H. Su Z. Huang Z. . (2025). Reciprocal relationships among problematic social media use, depression, and social anxiety of Chinese university students: disentangling between- and within-person effects. Addict. Behav. 172:108515. doi: 10.1016/j.addbeh.2025.108515 41056673

[ref42] Roberts S. O. Bareket-Shavit C. Dollins F. A. Goldie P. D. Mortenson E. (2020). Racial inequality in psychological research: trends of the past and recommendations for the future. Perspect. Psychol. Sci. 15, 1295–1309. doi: 10.1177/1745691620927709, 32578504

[ref43] Rohrer J. M. (2018). Thinking clearly about correlations and causation: graphical causal models for observational data. Adv. Methods Pract. Psychol. Sci. 1, 27–42. doi: 10.1177/2515245917745629

[ref44] Shi D. Maydeu-Olivares A. (2019). The effect of estimation methods on SEM fit indices. Educ. Psychol. Meas. 80, 421–445. doi: 10.1177/0013164419885164, 32425213 PMC7221491

[ref45] Sobih Z. M. Soliman E. R. A. Hashem R. E. M. Mohamed A. M. A. E. . (2025). Relationship between internet gaming disorder, impulsivity, and social anxiety disorder in a sample of university students. QJM 118: 627. doi: 10.1093/qjmed/hcaf224.145

[ref46] Steinberg L. Sharp C. Stanford M. S. (2013). New tricks for an old measure: the development of the Barratt impulsiveness scale–brief (BIS-brief). Psychol. Assess. 25, 216–226. doi: 10.1037/a0030550, 23148649

[ref47] Svetina D. Rutkowski L. Rutkowski D. (2019). Multiple-group invariance with categorical outcomes using updated guidelines: an illustration using Mplus and the lavaan/semTools packages. Struct. Equ. Model. 27, 111–130. doi: 10.1080/10705511.2019.1602776

[ref48] Van Borkulo C. D. Van Bork R. Boschloo L. Kossakowski J. J. Tio P. Schoevers R. A. . (2023). Comparing network structures on three aspects: a permutation test. Psychol. Methods 28, 1273–1285. doi: 10.1037/met0000476, 35404628

[ref49] Volkow N. D. Wise R. A. Baler R. (2017). The dopamine motive system: implications for drug and food addiction. Nat. Rev. Neurosci. 18, 741–752. doi: 10.1038/nrn.2017.130, 29142296

[ref50] Weber S. J. Kawa A. B. Beutler M. M. Kuhn H. M. Moutier A. L. Westlake J. G. . (2025). Dopamine transmission at D1 and D2 receptors in the nucleus accumbens contributes to the expression of incubation of cocaine craving. Neuropsychopharmacology 50, 461–471. doi: 10.1038/s41386-024-01992-2, 39300272 PMC11632087

[ref51] Wu M. J. Zhao K. Fils-Aime F. (2022). Response rates of online surveys in published research: a meta-analysis. Comput. Hum. Behav. Rep. 7, 1–11. doi: 10.1016/j.chbr.2022.100206

[ref52] Xu F. Cui W. Lawrence P. J. (2019). The intergenerational transmission of anxiety in a Chinese population: the mediating effect of parental control. J. Child Fam. Stud. 29, 1669–1678. doi: 10.1007/s10826-019-01675-3

[ref53] Yam C. W. Pakpour A. H. Griffiths M. D. Yau W. Y. Lo C. L. M. Ng J. M. T. . (2019). Psychometric testing of three Chinese online-related addictive behavior instruments among Hong Kong university students. Psychiatry Q. 90, 117–128. doi: 10.1007/s11126-018-9610-7 30328020

