# Supplementary material for: Cross-cultural adaptation and validation of the Chinese version of the Reward Deficiency Syndrome Questionnaire (RDSQ-29)
Source: Front Psychol. 2026 Oct 8;17:1929198. doi: 10.3389/fpsyg.2026.1929198 (PMC13687346; doi:10.3389/fpsyg.2026.1929198)
Supplement: Supplementary file 1 [file Table_1.pdf]

Additional file 1

Appendix 1: Original and Chinese-translated items of the RDSQ-29

Table A1. Translation comparison of RDSQ-29 items

**Original Item**	**Chinese Translation**
Activity	活跃倾向
1. I'm almost always active.	1. 我通常感到精力充沛，充满活力。
2. I like to be always active.	2. 我偏好保持忙碌和身体 / 精神活跃的状态。
3. I cannot stand inactivity.	3. 长时间无所事事会让我感到烦躁或不适。
4. If nothing special happens during the day, I feel empty and bored.	4. 如果一天中没有发生特别或有趣的事情，我会感到空虚或无聊。
5. Being inactive really annoys me.	5. 缺乏活力和新鲜感会让我感到特别沮丧。
Lack of Sexual Satisfaction	亲密关系体验
6. I can never get enough sex.	6. 即使在稳定的亲密关系中，我也常常感到不够满足或渴望更多。
7. I regularly change my sexual partners.	7. 我倾向于在较短时间内更换恋爱或亲密伴侣。
8. It can happen that I have more than one sexual partner at once.	8. 我有时会同时与不止一位伴侣保持情感或身体上的亲密关系。
Social Concerns	亲友关切

**Original Item**	**Chinese Translation**
9. My friends and family often worry about my lifestyle.	9. 我的朋友或家人曾对我的生活方式（如过度玩乐、冒险行为或作息不规律）表示担忧。
10. My friends or my family warned me several times that I overdo my recreational activities.	10. 我的亲友曾提醒或批评我参与的活动过于刺激或冒险。
Risk-Seeking Behavior	追求刺激行为
11. I like activities that' ll give me an adrenaline rush.	11. 我主动寻求能让我心跳加速、感到极度兴奋或刺激的活动。
12. Others would consider my activities dangerous.	12. 我乐于参与在他人看来可能具有较高风险的活动。
13. I like experimenting with extreme sports.	13. 我热衷于尝试极限运动（如蹦极、攀岩、滑板等） 或类似的高刺激体验。
14. Extreme sports stimulate me.	14. 极限运动或高风险活动能给我带来强烈的兴奋感和满足感。
15. I look for extreme challenges in my work, sports, or anything else.	15. 无论在学业、工作、运动还是爱好上，我都倾向于选择具有高挑战性或需要追求极致表现的任务。
General Reward Deficiency	日常满足感不足
16. I don' t receive gratification from everyday life.	16. 我觉得日常生活通常比较平淡，难以从中获得强烈的满足感或乐趣。

Original Item	Chinese Translation
17. While doing a task or work, I find myself already planning the next task.	17. 在进行一项任务或活动时，我常常会分心思考接下来要做的新鲜或更有趣的事情。
18. I consistently seek new situations and adventures.	18. 我持续渴望体验新事物和寻求新的冒险经历。
19. I prefer being active when going out with friends rather than just talking with each other.	19. 在社交聚会中，比起纯粹的聊天，我更倾向于组织或参与动态的活动（如运动、游戏、外出探险）。
20. I desire to participate in all aspects of life no matter the limits.	20. 我对尝试生活中各种可能带来强烈体验的事物抱有强烈兴趣，不愿过多限制自己。
21. I’ve tried many sports in my life.	21. 我尝试过多种不同类型且通常具有一定强度或刺激性的运动或活动。
22. I need more stimulation than others.	22. 与周围大多数人相比，我需要更强烈的感官刺激或新奇体验才能觉得某件事有趣。
23. I need more excitement than others.	23. 与周围大多数人相比，我需要体验到更高程度的兴奋感才能感到满足。
24. Most people think I can’ t sit still.	24. 我很难长时间保持安静或坐着不动。
25. I like to live dangerously.	25. 我主动追求能带来强烈刺激感或具有挑战性的生活方式和活动。

Original Item	Chinese Translation
26. I often want a good time no matter what I have to do to get it.	26. 为了追求当下的兴奋或快乐体验，我有时会低估或忽略其潜在的风险或负面后果。
27. Often I want to feel stimulated no matter what I have to do to get it.	27. 在追求强烈刺激感的过程中，其他因素（如成本、安全、责任）有时会被我暂时置于次要位置。
28. No pain or tiredness can deter me from doing something that I am passionate about.	28. 对于我真正热爱并投入的事情（即使它们消耗大量精力），我通常能展现出很强的坚持性和专注力。
29. When I' m doing something pleasurable, I can hardly stop myself.	29. 一旦开始从事我特别感兴趣的活动（如打游戏、刷剧、运动等），我常常难以控制停止的时间，容易沉浸其中。
